# From Full Spectra to Compact Signatures: Kolmogorov-Arnold Network-Based Hyperspectral Authentication of Dried Fish Maw

**DOI:** 10.3390/bios16060315

**Published:** 2026-06-01

**Authors:** Yuyan Xia, Yurong She, Xingguo Tian, Huadong Zeng

**Affiliations:** 1 School of Materials and New Energy, South China Normal University, Shanwei 516625, China; 2 College of Food Science, South China Agricultural University, Guangzhou 510642, China

**Keywords:** fish maw, hyperspectral imaging, Kolmogorov-Arnold Network, wavelength selection, nondestructive authentication

## Abstract

The authentication of fish maw is of considerable importance for preventing product substitution and protecting market confidence in high-value aquatic foods. This study developed a rapid and nondestructive authentication strategy by combining hyperspectral imaging (HSI) with wavelength selection and a Kolmogorov–Arnold Network (KAN) to discriminate 10 commercially representative fish maw varieties. Hyperspectral datasets were collected in the visible and near-infrared (VNIR, 400–1000 nm) and short-wave infrared (SWIR, 900–1700 nm) regions. To improve spectral quality and model robustness, four preprocessing methods (SG, SG−MeanNor, SG−DT, and SG−SNV) were evaluated, followed by the construction of PLS-DA, SVM, MLP, CNN, and KAN models. Feature wavelengths were subsequently selected separately from the VNIR and SWIR spectra using CARS, iVISSA, and SPA to establish reduced-variable authentication models. The results showed that SG-DT achieved the best overall preprocessing effect, confirming its ability to reduce spectral noise and baseline variation. In addition, SWIR-based models consistently outperformed VNIR-based models, suggesting that compositional information captured in the SWIR region played an important role in fish maw authentication. Among all tested models, the SWIR@SG-DT-SPA-KAN model exhibited the best performance, achieving 98.67% accuracy, 98.75% precision, 98.67% recall, and 98.64% F1-score using only 16 SPA-selected wavelengths from the SG-DT-preprocessed SWIR spectra. This study demonstrates that HSI coupled with feature wavelength and KAN modeling can provide an accurate and efficient tool for fish maw authentication. More importantly, the reduced-wavelength model offers practical potential for developing fast and cost-effective multispectral systems for authenticity screening in the aquatic food market.

## 1. Introduction

Fish maw, a dried marine product processed from fish swim bladders, is highly valued in Asian markets, particularly in China, because of its rich collagen content and perceived nutritional benefits [1,2,3]. The commercial value of fish maw is closely associated with the biological origin of the raw material, and products derived from Sciaenidae species are generally regarded as premium commodities because of their relative scarcity and high consumer acceptance [4]. With the continuous expansion of the fish maw trade, authenticity-related problems, including species substitution, misleading labeling, and quality fraud, have become increasingly prominent [5,6]. Previous studies have reported high rates of mislabeling in commercial fish maw products, with low-value species frequently being sold as high-value croaker-derived fish maw [7,8]. Such fraudulent practices not only undermine consumer confidence and market order but also pose challenges to product regulation and fishery resource conservation. Therefore, the development of a rapid and accurate authentication method for fish maw has considerable practical significance.

Authentication of fish maw is particularly challenging because the original biological characteristics of swim bladders are substantially altered during processing, including cutting, stretching, dehydration, and storage [1,6]. As a result, traditional morphology-based identification relying on appearance and experience is often unstable and subjective. DNA barcoding and other molecular approaches have been widely applied to fish maw and seafood species authentication and generally provide high identification accuracy [9]. However, these methods usually require destructive sampling and often involve complicated sample preparation, long analysis time, and relatively high cost [10], which limit their suitability for rapid and large-scale screening in commercial circulation and regulatory scenarios. Consequently, there is a strong need for nondestructive authentication techniques capable of capturing intrinsic compositional differences among fish maw products.

Hyperspectral imaging (HSI) combines continuous spectral information with spatial characterization information, enabling the nondestructive and simultaneous acquisition of surface features and chemical composition information of samples, thus showing broad application prospects in fields such as food quality evaluation, adulteration detection, and authenticity identification [11,12]. Compared with traditional appearance discrimination methods based on color, shape, or texture, the prominent advantage of HSI lies in its ability to provide discriminative information related to molecular vibrations and chemical composition differences, making it more suitable for processed foods with unstable appearance characteristics but differences in internal composition [13,14]. In the research on aquatic products, HSI has been successfully applied to tasks such as freshness evaluation, species identification, substitution detection, and quality grading [15,16], indicating that this technology can effectively capture discriminant information related to the biochemical composition and tissue structure of samples. For fish maw, this technical advantage is particularly important: fish maw is a highly processed dried product, and its original biological characteristics have been significantly weakened during the processing. Samples from different sources often have high similarity in appearance, while the differences in their collagen, residual moisture, lipids, and other trace components are more likely to be reflected in the spectral response. Therefore, compared with traditional appearance identification, HSI has greater potential to objectively distinguish the source of fish maw from the perspective of the internal physical and chemical composition of samples [17,18]. It should be clarified that currently, the research on spectral-based identification of fish maw authenticity is generally scarce at home and abroad, and there have been few dedicated HSI studies targeting fish maw authentication. In particular, the systematic comparison of VNIR and SWIR hyperspectral data for fish maw authentication remains largely unexplored. Notably, different wavebands have different sensitivities for characterizing information such as pigments, proteins, moisture, and lipids in fish maw samples, while there is a lack of relevant research to clarify the adaptability of the two wavebands in fish maw identification. Therefore, it is necessary to further compare their applicability and advantages in fish maw identification to fill the research gap in this field.

In the analysis of high-dimensional and complex spectral data, the selection of classification models is also crucial. Although traditional chemometric methods and conventional machine learning models have been widely used in spectral classification tasks, their feature expression ability and generalization performance may be limited when dealing with data with subtle differences between classes, strong feature nonlinearity, and overlapping information [19,20]. The Kolmogorov-Arnold Network (KAN) is a new type of neural network framework proposed in recent years, which realizes flexible characterization of complex nonlinear relationships between input variables by introducing learnable one-dimensional nonlinear functions on the edges of the network [21]. Compared with traditional multi-layer perceptrons, KAN has stronger expressive ability and better interpretability potential when modeling complex nonlinear mappings and has recently shown good application prospects in spectroscopic analysis tasks [22,23]. Considering the complex sources of fish maw samples, the weakened appearance information caused by processing, and the possible local overlap of spectral features between different categories, KAN may help capture the nonlinear relationships between hyperspectral variables and class labels, thereby improving the accuracy and robustness of fish maw authenticity identification.

**Figure 1:**
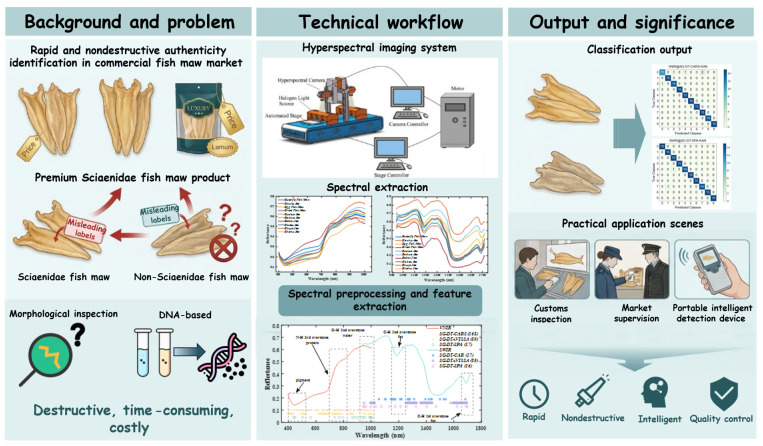
Research Framework for Fish Maw Classification Using VNIR/SWIR Hyperspectral Data and KAN Model.

Therefore, this study selected 10 commercially representative fish maw varieties to construct a sample system covering typical Sciaenidae fish maw and common non-Sciaenidae alternative fish maw. Hyperspectral image data of the samples in the VNIR and SWIR wavebands were collected to systematically compare the performance of different spectral preprocessing methods, characteristic wavelength screening strategies, and classification models in fish maw classification, with a focus on evaluating the application potential of KAN in this task. The overall framework is shown in Figure 1. This study aims to establish an intelligent identification method suitable for rapid and non-destructive identification of fish maw and provide technical support for the authenticity supervision of high-value dried marine products.

## 2. Materials and Methods

### 2.1. Sample Preparation

**Figure 2:**
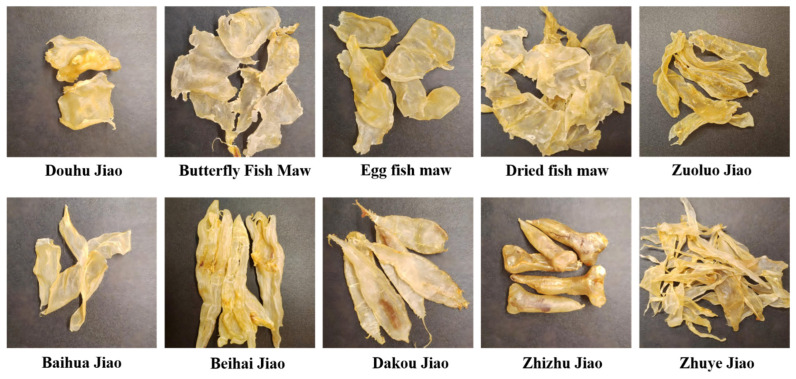
Fish maw sample images, first row non-Sciaenidae families, second row Sciaenidae families.

All fish maw samples used in this study were purchased from the Magong Aquatic Products Wholesale Market in Shanwei City, Guangdong Province, China. A total of 10 commercially available fish maw varieties were collected. Based on the biological classification of their parent species, these 10 fish maw varieties were divided into two categories: 5 varieties from the Sciaenidae family and the other 5 varieties from non-Sciaenidae families, as shown in Figure 2. For each of the 10 varieties shown in the figure, 50 independent samples with complete morphology were randomly selected, resulting in a total of 500 fish maw samples. After being transported back to the laboratory, all samples were individually numbered and sealed for storage in a light-proof, dry, temperature-controlled cabinet to ensure consistency in sample conditions during subsequent hyperspectral image acquisition.

### 2.2. Hyperspectral Imaging Systems and Data Extraction

**Figure 3:**
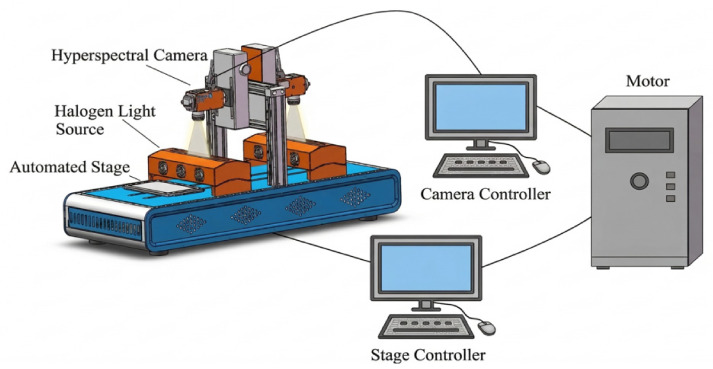
Hyperspectral imaging system.

The spectral data of fish maw samples were acquired using a hyperspectral imaging system. The system consisted of two hyperspectral cameras, FX10 and FX17 (SPECIM, Oulu, Finland), eight 35 W halogen lamps as illumination sources, a motorized translation stage, a computer for controlling the cameras and stage movement, and a dark enclosed chamber. Among them, the FX10 camera was used for the visible and near-infrared (VNIR) region, covering a wavelength range of 400–1000 nm with a spectral resolution of 5.5 nm, yielding a total of 224 bands. The FX17 camera was used for the short-wave infrared (SWIR) region, covering 900–1700 nm with a spectral resolution of 8 nm, also providing 224 bands. The hyperspectral imaging system used in this study is shown in Figure 3.

During image acquisition, the camera was mounted at a height of 32 cm above the translation stage and connected to a computer. Device connection and parameter settings were completed using Lumo Scanner software (2019-x64). Prior to sample scanning, white reference calibration was performed using a standard diffuse reflectance plate with a reflectance of 95%. The calibration plate fully covered the camera’s field of view, and the white reference image was acquired under static conditions. After calibration, each fish maw sample was placed on the translation stage for scanning. To avoid image stretching or compression, the stage moving speed was set to 9.8 mm/s. After scanning, each sample was immediately returned to a sealed bag to minimize quality changes before the next sample was collected.

After FX10 image acquisition was completed, the FX17 camera was installed, and the same calibration and scanning procedures were repeated. When the FX17 camera was used, the translation stage speed was adjusted to 7.5 mm/s to ensure proper image acquisition and spatial integrity. In this way, hyperspectral images of all fish maw samples were obtained in both the 400–1000 nm and 900–1700 nm spectral ranges.

After acquisition, the raw hyperspectral images in the 400–1000 nm and 900–1700 nm ranges were corrected using ENVI + IDL 4.8 software to eliminate errors caused by fluctuations in illumination intensity and other environmental factors. The correction was performed according to Equation (1).

(1) $$I_{C}=\frac{I-I_{D}}{I_{W}-I_{D}}$$

where I denotes the raw sample data, $I_{D}$ denotes the dark-reference data of the hyperspectral system, $I_{W}$ denotes the diffuse reflectance reference data used for calibration, and $I_{C}$ denotes the corrected sample data.

After radiometric correction, ROI extraction was performed to obtain the spectral information of each fish maw sample. A grayscale image with clear contrast between the sample and background was first selected from the corrected hyperspectral cube. Threshold segmentation was then used to generate a binary mask of the sample region, and isolated noise pixels were removed by morphological processing. The binary mask was applied to the corrected hyperspectral cube, and all valid pixels within the ROI were retained for spectral calculation. For each sample, the reflectance values of all pixels within the ROI were averaged at each wavelength to obtain one representative mean spectrum.

### 2.3. Dataset Split

To ensure the representativeness of the training and testing samples, this study employs the SPXY (sample set partitioning based on joint X-Y distances) method to partition the dataset. This method comprehensively considers the distribution characteristics of samples on the spectral variable (X) and its response variable (Y), achieving more uniform sample coverage in the feature space, thereby improving the stability and representativeness of the data partitioning [24]. Compared to random partitioning methods, SPXY effectively avoids bias problems caused by uneven sample distribution or information concentration.

In the specific implementation, the samples were first grouped according to fish maw varieties, and then the SPXY method was used to partition each variety. The ratio of the training set to the test set was set to 7:3, that is, 35 samples were selected from each variety as the training set and 15 samples as the test set. Finally, after merging the data from all varieties, a total of 350 samples were obtained for the training set and 150 samples for the test set.

To further evaluate model generalizability and reduce the dependence on a single train-test split, stratified k-fold cross-validation was additionally performed. In this study, a stratified 5-fold cross-validation strategy was adopted, in which samples from each fish maw variety were evenly distributed across the folds. In each iteration, four folds were used to train the model, and the remaining fold was used to evaluate its cross-validation performance. All preprocessing, wavelength selection, model training, and performance evaluation procedures were conducted independently within each training fold to avoid information leakage. The final cross-validation results were reported as the mean ± standard deviation of accuracy, precision, recall, and F1-score across the folds.

### 2.4. Preprocessing Methods

To reduce the impact of instrument noise, scattering effects, and baseline drift on spectral data and improve model stability and prediction accuracy, this study performed various preprocessing steps on the raw spectral data. The methods employed included Savitzky–Golay smoothing (SG), mean normalization (MeanNor), detrending (DT), and standard normal variable transformation (SNV).

SG smoothing primarily suppresses high-frequency noise while preserving spectral peak shape information as much as possible [25]. In this study, SG smoothing was performed using a window size of 11 points and a second-order polynomial fitting. Mean normalization reduces the impact of overall intensity differences among samples by scaling each spectrum according to its mean spectral intensity. Detrend processing eliminates baseline variations caused by instrument drift or sample surface inhomogeneities. Specifically, the DT method used in this study was polynomial detrending, in which a second-order polynomial baseline was fitted to each spectrum and then subtracted from the original spectrum. Standard normal variate transformation (SNV) normalizes the spectra, correcting for multiple scattering effects and path length differences, thereby enhancing the comparability between spectra.

In the specific implementation process, the original spectra were first smoothed using SG and then combined with different preprocessing methods to form three preprocessing strategies: SG-MeanNor, SG-DT, and SG-SNV.

### 2.5. Wavelength Selection Methods

To reduce redundant information and multicollinearity in hyperspectral data and to improve modeling efficiency and classification performance, three wavelength selection methods, namely CARS (Competitive Adaptive Reweighted Sampling), iVISSA (interval Variable Iterative Space Shrinkage Approach), and SPA (Successive Projections Algorithm), were employed to optimize the spectral variables in this study.

CARS is a variable selection method based on the principle of “survival of the fittest”. It repeatedly establishes calibration models through Monte Carlo sampling and evaluates the importance of each variable according to its contribution to the model. During the selection process, variables with low contributions are gradually eliminated, whereas variables with high contributions are preferentially retained. The optimal variable subset is then determined by cross-validation [26]. CARS is characterized by its ability to efficiently identify key wavelengths that are highly relevant to classification or modeling tasks from high-dimensional spectral data, and it has been widely used in spectral analysis because of its efficiency and practicality.

iVISSA is a wavelength selection method based on interval optimization. Unlike methods that directly select individual wavelengths, iVISSA divides the continuous spectrum into a number of candidate intervals and iteratively searches for the spectral regions that contribute most to model performance by progressively shrinking the variable space [24]. This method takes into account the continuity of hyperspectral data along the wavelength axis and is therefore particularly suitable for spectral datasets in which useful information is distributed over continuous wavelength intervals. By preserving locally informative regions while reducing the influence of redundant variables, iVISSA can improve both the stability and interpretability of the selected features.

SPA is a forward variable selection method designed to minimize collinearity among variables [27]. Its main idea is to successively select variables that provide the largest amount of new information while avoiding redundancy with variables already selected. In this way, SPA can effectively reduce multicollinearity in spectral data and generate a compact subset of representative wavelengths. Compared with other selection methods, SPA generally retains fewer variables, resulting in simpler model structures, higher computational efficiency, and improved interpretability.

### 2.6. Kolmogorov–Arnold Networks

We adopt the Kolmogorov–Arnold Network (KAN) as the backbone of the proposed classifier. In contrast to conventional multi-layer perceptrons (MLPs), which apply fixed nonlinear activations at the nodes, KAN parameterizes learnable univariate nonlinear transformations on the edges, while retaining summation-only operations at the nodes.

Let $x=[x_{1},x_{2},\ldots ,x_{n}{]}^{⊤}\in R^{n}$ denote the input spectral vector of a fish maw sample, where $x_{p}$ is the reflectance value at the p-th wavelength and n is the number of spectral bands. According to the Kolmogorov–Arnold representation theorem, a multivariate continuous mapping can be represented as a finite composition of univariate functions and additions. Accordingly, the spectral classification function can be written as:

(2) $$f\left(x\right)=\sum_{q=1}^{2n+1}\Phi_{q}\left(\sum_{p=1}^{n}\phi_{q,p}\left(x_{p}\right)\right)$$

where $\phi_{q,p}(\cdot )$ denotes a learnable univariate transformation and $\Phi_{q}(\cdot )$ denotes an outer aggregation function.

For a deep KAN, given the input feature vector $x\in R^{d_{l}}$ at the l-th layer, the i-th output of the $\left(l+1\right)$-th layer is defined as:

(3) $$x_{i}^{l+1}=\sum_{j=1}^{d_{l}}\phi_{i,j}^{l}\left(x_{j}^{l}\right)$$

where $\phi_{i,j}^{\left(l\right)}\left(\cdot \right)$ is the learnable edge function associated with the connection from the j-th input dimension to the i-th output dimension.

In this study, each edge function is parameterized as:

(4) $$\phi \left(u\right)=wu+\sum_{k=1}^{K}c_{k}B_{k}\left(u\right)$$

where w is the coefficient of the base term, $B_{k}(u)$ is the k-th B-spline basis function, and $c_{k}$ is its corresponding learnable coefficient. This parameterization combines a low-order base mapping with a spline-based nonlinear correction, enabling the model to capture both smooth global trends and localized spectral variations. The detailed implementation of the KAN block and the overall architecture are provided in Table 1 and Table 2.

The input dimension of the network equals the number of wavelengths, and the output dimension equals the number of fish maw classes. In the KANLinear layer, the spline branch uses a grid size of 5 and a spline order of 3. The model is trained using the cross-entropy loss and the Adam optimizer, with an initial learning rate of $1\times 10^{-3}$ and a weight decay of $1\times 10^{-4}$. A StepLR scheduler is employed with a step size of 30 and a decay factor γ=0.5. The batch size is set to 32.

### 2.7. Comparison Models

To evaluate the performance of the proposed model, partial least squares discriminant analysis (PLS-DA), support vector machine (SVM), multilayer perceptron (MLP) and Convolutional Neural Network (CNN) were used for comparison [28,29,30]. The main hyperparameters are listed in Table 3.

For PLS-DA, the number of latent variables was optimized independently under each preprocessing and wavelength-selection condition. Candidate latent variables ranging from 10 to 15 were tested, and the value yielding the best validation performance was selected for each model. For SVM, the radial basis function (RBF) kernel was employed. The hyperparameters, including the penalty parameter C and kernel parameter γ, were optimized using a grid search with cross-validation within the training set. Specifically, C was searched within [0.1, 1, 10, 100], and γ was selected from [“scale”, 0.001, 0.01, 0.1, 1]. The optimal combination was determined as the one achieving the highest cross-validation accuracy within the training set, and the independent test set was used only for final model evaluation. The MLP model adopted a two-layer fully connected structure (256 and 128 neurons), and the dropout rate was set to 0.2. All models were trained and evaluated under identical data preprocessing and data partitioning conditions.

### 2.8. Computational Environment

All model construction and evaluation procedures were implemented in Python 3.9. Conventional machine learning models, including PLS-DA and SVM, were implemented using scikit-learn, while MLP, CNN, and KAN were implemented using PyTorch 2.11. All experiments were conducted on a workstation equipped with an NVIDIA GeForce RTX 3060 GPU. The same software environment and data partitioning strategy were used for all models to ensure fair comparison.

### 2.9. Model Interpretation

SHAP (Shapley Additive Explanations) is a model interpretation method derived from cooperative game theory [31]. Its main idea is to quantify the contribution of each input variable to the model output by treating each feature as a “player” in a cooperative game and assigning an importance value based on its marginal contribution across different feature combinations. In spectral analysis, each wavelength can be regarded as an individual feature influencing the model prediction. SHAP evaluates how the inclusion of a specific wavelength changes the prediction under different feature subsets, thereby estimating its contribution to the final classification or regression result. Unlike conventional feature importance methods that only provide an overall ranking, SHAP can reveal both the global importance of wavelengths across the dataset and the positive or negative effect of each wavelength on the prediction of an individual sample. Therefore, the introduction of SHAP into hyperspectral modeling helps identify the most informative spectral regions from a large number of continuous bands and provides a more physically meaningful interpretation of model decisions when combined with the absorption characteristics or chemical bond assignments associated with specific wavelengths.

### 2.10. Evaluation Metrics

The performance of all models was evaluated using accuracy, precision, recall, and F1-score, which were defined by Equations (5)–(8):

(5) $$Accuracy=\frac{TP+TN}{TP+TN+FP+FN}$$

(6) $$Precision=\frac{TP}{TP+FP}$$

(7) $$Recall=\frac{TP}{TP+FN}$$

(8) $$F1-score=\frac{2\times Precision\times Recall}{Precision+Recall}$$

These metrics were calculated based on the confusion matrix, where true positives (TP), true negatives (TN), false positives (FP), and false negatives (FN) denote the number of correctly and incorrectly classified samples.

## 3. Results

### 3.1. Spectral Analysis

**Figure 4:**
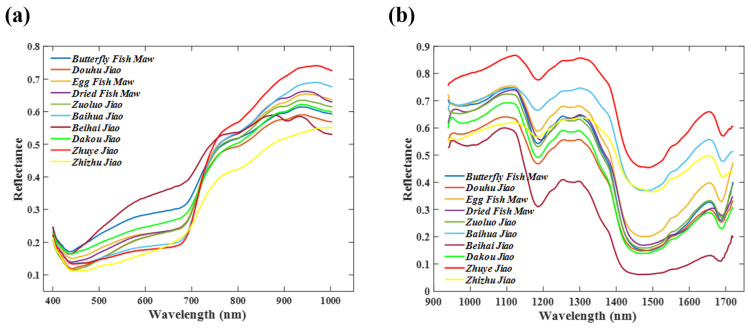
Average reflectance spectra of ten fish maw varieties in the (**a**) VNIR and (**b**) SWIR ranges.

Figure 4 shows the average reflectance spectra of 10 different varieties of fish maw in the VNIR and SWIR bands. Overall, the samples showed a relatively consistent spectral evolution trend in both bands, indicating that the different fish maws have strong similarities in their basic chemical composition, mainly related to collagen, water, and a small amount of inorganic components.

In VNIR, the reflectance of the samples in the visible light region (400–700 nm) was generally low and showed a gradual upward trend with increasing wavelength, which is mainly related to the sample surface color, gloss characteristics, and diffuse reflection behavior [32]. After entering the near-infrared region, the curve gradually flattens out and a slight absorption appears near 950–1000 nm, which may be related to the overtone absorption of O-H bonds in water and N-H bonds in proteins [33].

In contrast, the SWIR band exhibits more pronounced molecular vibrational absorption characteristics, with a broad and deep absorption valley near 1450 nm, mainly attributed to the first overtone of the O-H bond stretching vibration [34]; the weak absorption near 1150–1200 nm is related to the second overtone of the C-H bond [35], while the persistent low reflectance in the 1500–1700 nm region may be related to the overlapping absorption of the first overtones of the N-H and C-H bonds [36]. Although the overall spectral profiles of different fish maw varieties were similar, their reflectance baselines still show significant differences, indicating that different varieties differ in tissue density and internal structure.

### 3.2. Preprocessing of Spectral Data

**Figure 5:**
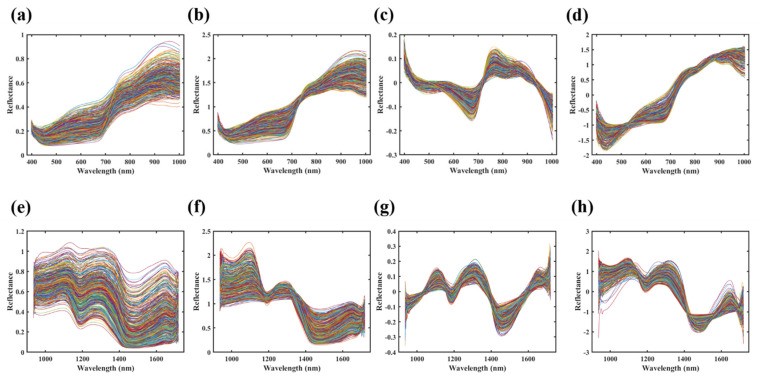
Spectra in the VNIR and SWIR regions after different preprocessing methods. (**a**) VNIR-SG, (**b**)VNIR-SG-MeanNor, (**c**) VNIR-SG-DT, (**d**) VNIR-SG-SNV, (**e**) SWIR-SG, (**f**) SWIR-SG-Mea-Nor, (**g**) SWIR-SG-DT, (**h**) SWIR-SG-SNV.

Figure 5 presents the spectral profiles after different preprocessing methods. It can be observed that SG smoothing effectively reduces random noise and yields smoother spectral curves, although baseline shifts and scattering variations among samples still remain. Based on this, SG-MeanNor normalization further reduces intensity differences across samples, resulting in a more compact distribution of spectra. SG-Detrend mainly removes baseline drift, causing the spectra to fluctuate around zero and enhancing local feature variations. In comparison, SG-SNV shows a more pronounced effect in correcting scattering, not only improving spectral consistency but also enhancing comparability among samples. Subsequently, both raw spectra and preprocessed spectra were used as inputs for full-wavelength PLS-DA, SVM, MLP, and KAN models to evaluate the impact of different preprocessing strategies on classification performance.

### 3.3. Model Performance Using Full Wavelengths

**Figure 6:**
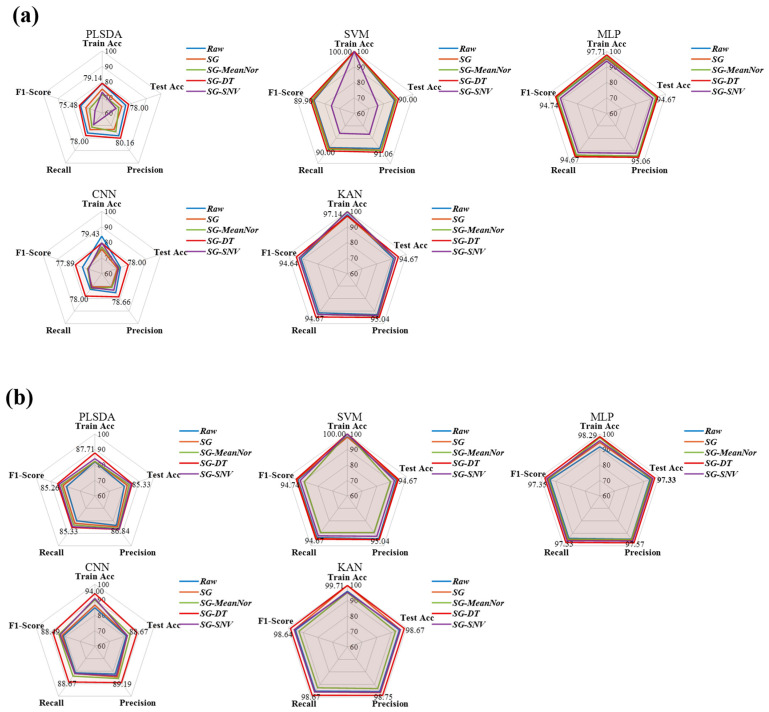
Radar chart comparison of classification performance of different models using the optimal preprocessing method (SG-DT), (**a**) VNIR, (**b**) SWIR.

Table S1 summarizes the classification performance of different preprocessing methods combined with classification models in the VNIR and SWIR ranges. Figure 6 further presents the multi-index performance of the optimal models selected under each spectral range and preprocessing condition. Overall, preprocessing had a clear influence on model performance, and this effect showed relatively consistent trends across different spectral ranges and modeling methods. To further examine the class-wise recognition behavior of the selected models, the confusion matrices of the optimal full-spectrum models are shown in Figure S1.

First, the single preprocessing method SG shows a positive effect in most models. Compared to the original spectra, after SG processing, the test accuracies of SVM, MLP, and KAN in the VNIR band increased from 87.33%, 93.33%, and 91.33% to 88.00%, 94.00%, and 92.67%, respectively; and in the SWIR band, they increased from 93.33%, 94.00%, and 95.33% to 94.00%, 95.33%, and 96.00%, respectively. This phenomenon indicates that moderate smoothing can effectively suppress high-frequency random noise, improve spectral signal quality, and thus enhance the model’s ability to identify class differences. In other words, the improvement results of SG provide a reasonable basis for further constructing composite preprocessing. However, the performance gain from relying solely on SG smoothing is still limited and does not reach the optimal level across all model combinations. After further introducing detrending, SG-DT achieved the best test accuracy for all evaluated models in both VNIR and SWIR ranges. In the VNIR range, the test accuracies of PLSDA, SVM, MLP, CNN, and KAN after SG-DT preprocessing reached 78.00%, 90.00%, 94.67%, 78.00%, and 94.67%, respectively. In the SWIR range, the corresponding values further increased to 85.33%, 94.67%, 97.33%, 88.67%, and 98.67%, respectively. These results suggest that baseline drift and slowly varying background interference are important factors affecting spectral classification. The combination of SG and DT can reduce high-frequency noise while weakening trend-related variations, thereby retaining more useful discriminative spectral information. The results of the CNN model deserve particular attention. Although CNN benefited from SG-DT preprocessing, its overall performance was lower than that of SVM, MLP, and KAN. The best CNN accuracies were only 78.00% in VNIR and 88.67% in SWIR, which were clearly lower than those of KAN under the same preprocessing conditions. This indicates that conventional convolutional structures do not necessarily provide an advantage in limited-sample one-dimensional spectral classification. CNN models usually require sufficient samples to learn stable local patterns, whereas the current dataset may favor models that can handle compact spectral inputs and limited sample sizes more effectively, such as SVM, MLP, and KAN.

From the spectral-range perspective, SWIR-based models generally achieved better classification performance than VNIR-based models, particularly in nonlinear models such as MLP and KAN. This difference may be attributed to the distinct spectral information provided by the two wavelength ranges. VNIR spectra are mainly affected by surface-related optical characteristics, such as color, texture, and scattering, which may be insufficient for distinguishing fish maw samples with similar appearances and overlapping spectral profiles. In contrast, SWIR spectra contain more pronounced molecular vibration information, including overtones and combination bands related to O–H, C–H, and N–H groups, which are closely associated with water status, collagen/protein structure, and intrinsic compositional differences in fish maw. Therefore, SWIR can provide more chemically relevant and class-specific information for classification. The lower performance of PLSDA in both ranges further suggests that the spectral differences among fish maw samples may not be fully described by linear projections, whereas nonlinear models such as SVM, MLP, and KAN are more capable of capturing subtle and nonlinear spectral variations, especially in the SWIR region.

Among all combinations, KAN combined with SG-DT preprocessing in the SWIR range achieved the best overall performance, with test accuracy, precision, recall, and F1-score of 98.67%, 98.75%, 98.67%, and 98.64%, respectively. Compared with the conventional CNN under the same spectral range and preprocessing strategy, the accuracy of KAN was improved by 10.00 percentage points. This result indicates that the SWIR-SG-DT-KAN combination was more effective in capturing discriminative spectral information from fish maw samples under the current sample size.

### 3.4. Selection of Key Wavelengths

**Figure 7:**
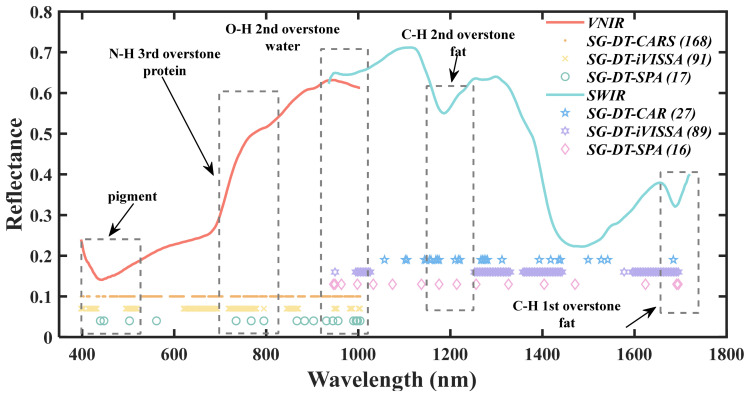
The distribution of key wavelengths selected by different wavelength selection methods.

Based on the optimal preprocessing methods identified from full-spectrum modeling, key wavelength extraction was further performed. The specific wavelength information is listed in Tables S2 and S3, and their distribution is shown in Figure 7. Overall, different wavelength selection methods were able to capture the major informative regions related to sample classification in both the VNIR and SWIR ranges, but clear differences were observed in the number and distribution pattern of the selected wavelengths.

In the VNIR region, CARS selected the largest number of feature wavelengths, with a total of 168 bands distributed broadly across the entire spectral range, showing a strong global retention pattern. This indicates that CARS tends to retain a relatively large number of variables contributing to the model, thereby preserving as much useful information from the original spectra as possible. In contrast, iVISSA and SPA selected 91 and 17 bands, respectively. Although the number of variables was markedly reduced, the selected wavelengths were still mainly concentrated in several key spectral intervals, suggesting that these two methods place greater emphasis on extracting locally representative variables while reducing dimensionality. As shown in Figure 7, the feature wavelengths in the VNIR region were mainly distributed in the 400–550 nm and 680–1000 nm ranges. These regions may be associated with pigment absorption, protein-related N-H higher-order overtone absorption, and water-related O-H overtone absorption, indicating that the selected wavelengths may have certain physicochemical relevance.

In the SWIR region, the differences among the three methods became more pronounced. CARS selected only 27 bands, which were substantially fewer than in the VNIR region, and these bands were mainly concentrated in a few key absorption regions, indicating that the informative features contributing to classification were more concentrated in this spectral range. By comparison, although iVISSA and SPA selected 89 and 16 bands, respectively, their feature wavelengths were mainly distributed across multiple key absorption intervals, showing stronger interval representativeness. Specifically, these wavelengths were mainly located around 950–1000 nm, 1100–1300 nm, 1400–1450 nm, and 1550–1700 nm. These regions are generally associated with water-related O-H overtone absorption and lipid-related second and first overtone absorptions of C-H, suggesting that the selected variables in the SWIR region may more directly reflect differences in the chemical composition of fish maw samples.

Overall, the differences in the number and distribution pattern of selected wavelengths among the various methods reflect different trade-offs between information retention and redundancy reduction, resulting in variable subsets with distinct structural characteristics. This also provides support for the ability of subsequent models to maintain relatively high classification performance under low-dimensional input conditions.

### 3.5. Model Performance Using Key Wavelengths

**Figure 8:**
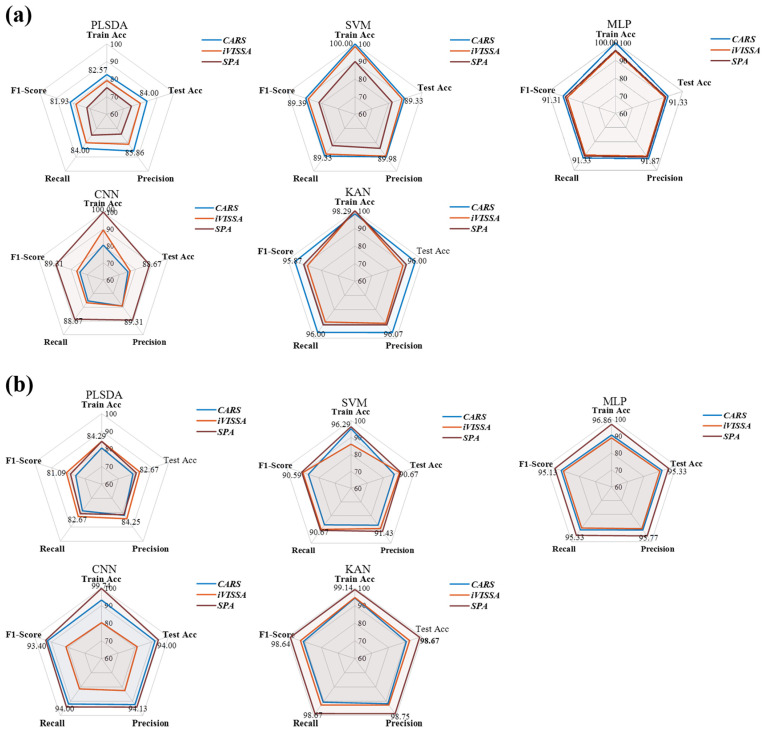
Radar chart of the best-performing models obtained using wavelength selection methods in the VNIR and SWIR regions. The figure compares Accuracy, Precision, Recall, and F1-score to illustrate the overall classification performance of the optimal reduced-variable models. (**a**) VNIR, (**b**) SWIR.

Table S4 summarizes the classification performance of different models after wavelength selection in the VNIR and SWIR ranges, and the better-performing models are further visualized using radar plots in Figure 8. Overall, compared with full-spectrum modeling, the effect of wavelength selection was not consistent across all cases, but depended on the spectral range, wavelength selection method, and classification model. In the VNIR range, CARS showed the best overall performance among the three wavelength selection methods. After CARS-based wavelength selection, the test accuracies of PLSDA, SVM, MLP, and KAN reached 84.00%, 89.33%, 91.33%, and 96.00%, respectively. Compared with their best full-spectrum models, the accuracies of PLSDA and KAN increased from 78.00% and 94.67% to 84.00% and 96.00%, respectively, indicating that appropriate variable reduction in the VNIR range can remove redundant or noisy information and enhance the discriminative ability of effective spectral features. In contrast, the accuracies of SVM and MLP slightly decreased after wavelength selection, suggesting that some weak but complementary information distributed across the full spectrum may have been removed during variable selection. For the CNN model, the test accuracy reached 88.67% after SPA-based feature extraction in the VNIR range, indicating that selected wavelengths could also provide useful discriminative information for deep learning models, although its performance was still lower than that of KAN.

In the SWIR range, wavelength selection did not further improve the accuracy of most models beyond their corresponding best full-spectrum results. This may be because the SWIR full spectrum already contains relatively rich and continuous chemical information, and excessive variable reduction may weaken the contribution of some continuous absorption features to classification. Nevertheless, wavelength selection still showed clear value for model simplification. For example, the CNN model achieved a test accuracy of 94.00% after SPA-based feature extraction in the SWIR range, suggesting that a limited number of selected wavelengths could still support effective classification. More importantly, the combination of KAN and SPA achieved the same highest test accuracy as the best SWIR full-spectrum model, reaching 98.67%, while reducing the number of input variables to only 16. This result demonstrates that the key discriminative information in the SWIR range can be effectively represented by a small number of feature wavelengths. Therefore, for SWIR data, the main advantage of wavelength selection was not necessarily further accuracy improvement, but rather dimensionality reduction, removal of redundant variables, and simplification of the model input.

Overall, the role of wavelength selection varied among different spectral ranges and models. In the VNIR range, wavelength selection was more likely to improve model performance, especially for PLSDA and KAN. In the SWIR range, wavelength selection mainly contributed to data reduction and model simplification while maintaining high classification accuracy. The additional CNN results further showed that deep learning models can also extract useful discriminative information from selected wavelengths, but their overall performance was still inferior to that of KAN. Considering both full-spectrum and selected-wavelength modeling results, KAN maintained high accuracy and good stability under different input conditions, indicating its strong adaptability for nonlinear classification of fish maw hyperspectral data.

### 3.6. Cross-Validation Analysis of Selected Models

To further evaluate model generalizability, stratified 5-fold cross-validation was performed on representative models selected from the SPXY-based screening results. Since SWIR-SG-DT showed the best overall performance in the comprehensive model comparison, the cross-validation analysis focused on models under this optimal spectral-preprocessing condition. PLS-DA, SVM, MLP, CNN, and KAN were included to compare different classifier types, and the final reduced-wavelength SWIR-SG-DT-SPA-KAN model was further evaluated to verify the robustness of the proposed compact model.

As shown in Table S5, PLS-DA and SVM showed relatively lower cross-validation performance, with accuracies of 83.60 ± 3.21% and 86.40 ± 3.21%, respectively. CNN achieved a similar accuracy of 86.60 ± 3.05%, indicating that the convolutional model did not provide a clear advantage under the current sample-level spectral dataset. In contrast, MLP and KAN achieved higher and more stable performance, with accuracies of 95.80 ± 1.30% and 96.00 ± 1.41%, respectively. The final SWIR-SG-DT-SPA-KAN model achieved the best cross-validation performance, with an accuracy, precision, recall, and F1-score of 97.40 ± 1.82%, 97.58 ± 1.68%, 97.40 ± 1.82%, and 97.40 ± 1.81%, respectively. These results demonstrate that the reduced-wavelength KAN model maintained robust classification performance across different validation folds, confirming that its high accuracy was not dependent on a single train-test split.

### 3.7. Confusion Matrix Analysis of Optimal Models

**Figure 9:**
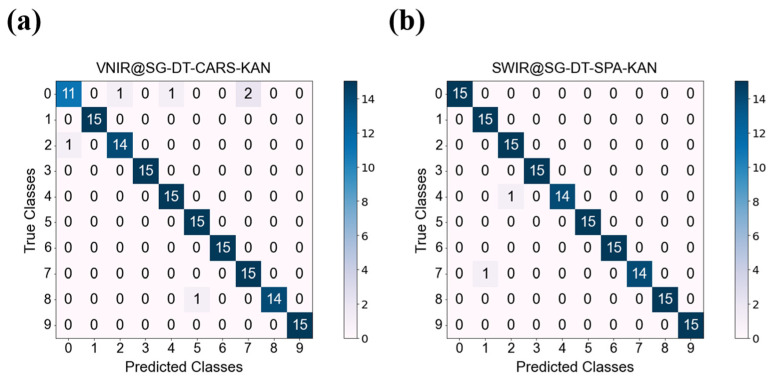
Confusion matrices of the optimal models, (**a**) VNIR, (**b**) SWIR. **Note**: 0: Butterfly fish maw; 1: Douhu Jiao; 2: Egg fish maw; 3: Dried fish maw; 4: Zuoluo Jiao; 5: Baihua Jiao; 6: Beihai Jiao; 7: Dakou jiao; 8: Zhuye Jiao; 9: Zhizhu Jiao.

As shown in Figure 9, both optimal models exhibit a pronounced diagonal pattern in the confusion matrices, indicating strong overall classification performance. However, noticeable differences can be observed in their class-level misclassification patterns. For VNIR@SG-DT-CARS-KAN, a relatively higher number of off-diagonal elements is present, with misclassifications mainly occurring among specific classes. For instance, some samples from Butterfly fish maw are misclassified as Egg fish maw, Zuoluo Jiao, and Dakou jiao. In addition, a small portion of Zhuye Jiao samples are misclassified as Baihua Jiao. These results suggest that, within the VNIR region, certain classes still exhibit overlapping discriminative information, leading to limited separability at local class boundaries.

In contrast, the confusion matrix of SWIR@SG-DT-SPA-KAN shows a more concentrated diagonal distribution with significantly fewer off-diagonal elements. Only minor misclassifications are observed between a few classes, such as between Zuoluo Jiao and Egg fish maw, and between Dakou jiao and Butterfly fish maw. This indicates that the SWIR-based model achieves clearer class boundaries and more stable classification performance. Notably, this model achieves the optimal classification results using only 16 SPA-selected wavelengths from the SWIR spectra, suggesting that a small number of key variables is sufficient to effectively characterize the essential differences among samples.

### 3.8. Analysis of SHAP Visualization Results

**Figure 10:**
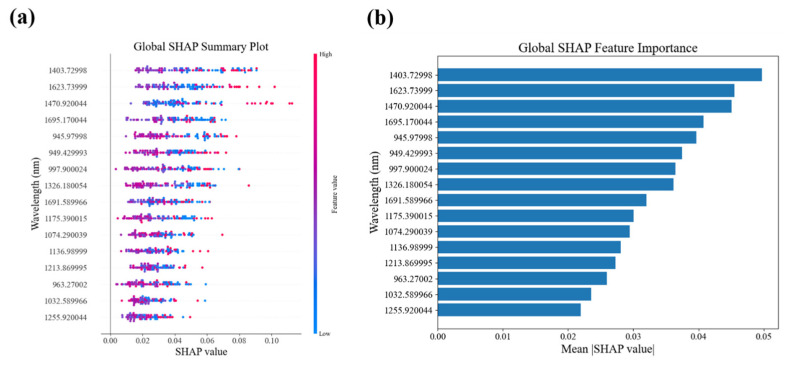
Global SHAP analysis of the reduced-wavelength KAN model for dried fish maw authentication: (**a**) SHAP summary plot; (**b**) SHAP feature importance plot.

The SHAP analysis revealed that the discrimination of dried fish maw was mainly driven by a compact set of informative wavelengths in the SWIR region. As shown in Figure 10, the most influential wavelengths were located at approximately 1403.73, 1470.92, 1623.74, and 1695.17 nm, with additional contributions from wavelengths between 945 and 1326 nm. These wavelengths are closely related to the overtone and combination vibrations of O–H, C–H, and N–H bonds, which are associated with the major biochemical constituents of dried fish maw, including residual water, collagen-rich proteins, and minor lipid components.

Specifically, the important wavelengths near 1403 and 1470 nm are located around the characteristic water absorption region near 1450 nm, which is mainly attributed to the first overtone of O–H stretching vibrations. Since dried fish maw is produced through dehydration of swim bladders, differences in residual moisture content, water-binding capacity, and matrix compactness may contribute to variations in this spectral region. The wavelengths around 1213 and 1326 nm may be associated with C–H second-overtone and combination absorption, reflecting differences in organic matrix composition and tissue structure among fish maw varieties. In addition, the highly ranked wavelengths near 1623 and 1695 nm may be related to the first overtone or combination vibrations of C–H and N–H bonds, which are closely linked to lipid residues and collagen/protein structures. Considering that collagen is the dominant component of fish maw, variations in collagen organization, protein conformation, and drying-induced structural differences may provide important biochemical information for species authentication.

Overall, the SHAP results suggest that the key wavelengths selected by SPA contributed strongly to model prediction, but were also closely related to the main biochemical characteristics of dried fish maw. The contributions of these wavelengths were mainly associated with moisture-related O–H absorption and collagen/protein-related C–H and N–H responses, which provide a reasonable explanation for the high performance of the reduced-wavelength KAN model.

## 4. Conclusions

This study developed a hyperspectral imaging-based strategy for the nondestructive authentication of fish maw samples. The results demonstrated that the SWIR region combined with composite preprocessing, wavelength selection, and nonlinear modeling enabled highly accurate classification of fish maw samples. Among all tested strategies, SWIR@SG-DT-SPA-KAN achieved the best performance, with an accuracy, precision, recall, and F1-score of 98.67%, 98.75%, 98.67%, and 98.64%, respectively, using only 16 SPA-selected wavelengths from the SWIR spectra. These findings indicate that a limited number of key wavelengths in the SWIR region can effectively characterize the major discriminative information of fish maw samples, while KAN shows strong capability in modeling complex nonlinear features. Therefore, hyperspectral imaging combined with wavelength selection and the KAN model provides an effective approach for the rapid, nondestructive, and intelligent classification of fish maw samples. However, the generalizability of the proposed method still needs further validation using fish maw samples from different regions, suppliers, processing batches, storage conditions, and imaging environments. Although KAN achieved better performance than MLP and CNN in this study, its advantages may depend on dataset size, selected wavelengths, and parameter settings, and further external validation is still required.

## Figures and Tables

**Table 1:** Overall procedure of the proposed KAN-based classifier.

Module	Description
Input	Input feature vector $x\in R^{D}$
KAN Block 1	Generate the first hidden representation $h^{\left(1\right)}\in R^{256}$ using the KAN block defined in Table 2
KAN Block 2	Generate the second hidden representation $h^{\left(2\right)}\in R^{128}$ using the KAN block defined in Table 2
Classifier	Apply a linear projection to obtain class logits: $z\leftarrow Linear(h^{\left(2\right)})$
Output	Output logits $z\in R^{C}$

Note: D denotes input data dimension, C represents the number of categories.

**Table 2:** Internal procedure of a KAN block.

Module	Description
Base mapping	Compute the base component by $u_{base}\leftarrow {Linear}_{base}(SiLU(u))$
Spline mapping	Compute the spline component by $u_{spline}\leftarrow {Linear}_{spline}(Flatten(\Phi (u))$
Feature fusion	Fuse the two components by element-wise summation: $h\leftarrow u_{base}+u_{spline}$
Normalization	Apply batch normalization: h←BatchNorm1d(h)
Activation	Apply nonlinear activation: h←ReLU(h)
Regularization	Apply dropout with *p* = 0.2: h←Dropout(h, p=0.2)
Output	Return the transformed feature representation h

Note: Φ(·) denotes the B-spline basis expansion, and Flatten(·) reshapes the expanded spline features before linear projection.

**Table 3:** Hyperparameter settings of comparison models.

Model	Settings
PLS-DA	Number of latent variables = 10–15
SVM	RBF kernel; C = 100; γ = 0.01
MLP	Hidden layers = (256, 128); Dropout = 0.2, Optimizer = Adam; LR = 1 × 10^−3^; Epochs = 100
CNN	Conv1D filters = 32–64–128; kernel sizes = 5–5–3; BatchNorm1D + ReLU; MaxPool1D + AdaptiveAvgPool1D; dropout = 0.3; Adam optimizer; learning rate = 1 × 10^−3^; weight decay = 1 × 10^−4^; batch size = 32; epochs = 120

## Data Availability

The data and code that support the findings of this study are available from the corresponding author upon reasonable request.

## Supplementary Materials

The following supporting information can be downloaded at: https://www.mdpi.com/article/10.3390/bios16060315/s1, Table S1. Classification performance of different models under various preprocessing methods in the VNIR and SWIR ranges. Table S2. Specific characteristic wavelengths selected by different feature selection methods in VNIR. Table S3. Specific characteristic wavelengths selected by different feature selection methods in SWIR. Table S4. Classification performance of different models based on wavelength selection methods in the VNIR and SWIR regions. Table S5. Stratified 5-fold cross-validation performance of selected models. Figure S1. Classification performance of different models using full-spectrum data with optimal preprocessing. (a) VNIR, (b) SWIR.

## Abbreviations

The following abbreviations are used in this manuscript:

HSI	Hyperspectral imaging
VNIR	Visible and near-infrared
SWIR	Short-wave infrared
KAN	Kolmogorov–Arnold Network
PLS-DA	Partial least squares discriminant analysis
SVM	Support vector machine
MLP	Multilayer perceptron
CNN	Convolutional neural network
SG	Savitzky–Golay smoothing
MeanNor	Mean normalization
DT	Detrending
SNV	Standard normal variate
CARS	Competitive adaptive reweighted sampling
iVISSA	interval Variable Iterative Space Shrinkage Approach
SPA	Successive projections algorithm
SPXY	Sample set partitioning based on joint X–Y distances
SHAP	Shapley Additive Explanations
RBF	Radial basis function
TP	True positive
TN	True negative
FP	False positive
FN	False negative
