# Supplementary material for: From Full Spectra to Compact Signatures: Kolmogorov-Arnold Network-Based Hyperspectral Authentication of Dried Fish Maw
Source: Biosensors (Basel). 2026 Jun 1;16(6):315. doi: 10.3390/bios16060315 (PMC13297219; doi:10.3390/bios16060315)
Supplement: Supplementary file 1 [file biosensors-16-00315-s001.zip › biosensors-4300776-supplementary.pdf]

Supplementary materials

Table S1. Classification performance of different models under various preprocessing methods in the VNIR and SWIR ranges.

Range	Model	Processing Method	Train (%)	Test (%)			
			Accuracy	Accuracy	Precision	Recall	F1-Score
VNIR	PLS-DA	Raw (Lvs=14)	79.14	76.00	78.22	76.00	74.56
		SG (Lvs=15)	75.43	73.33	73.15	73.33	71.07
		SG-MeanNor (Lvs=15)	72.00	71.33	75.03	71.33	68.48
		**SG-DT** (Lvs=12)	**79.14**	**78.00**	**80.16**	**78.00**	**75.48**
		SG-SNV (Lvs=10)	73.43	69.33	62.91	69.33	64.87
	SVM	Raw	100	87.33	88.06	87.33	87.27
		SG	100	88.00	89.31	88.00	87.82
		SG-MeanNor	100	88.67	89.59	88.67	88.71
		**SG-DT**	**100**	**90.00**	**91.06**	**90.00**	**89.90**
		SG-SNV	100	76.00	76.71	76.00	75.54
	MLP	Raw	95.14	93.33	94.19	93.33	93.43
		SG	95.43	94.00	94.55	94.00	94.04
		SG-MeanNor	96.57	93.33	94.17	93.33	93.45
		**SG-DT**	**97.71**	**94.67**	**95.06**	**94.67**	**94.74**
		SG-SNV	93.71	91.33	91.87	91.33	91.46
	CNN	Raw	84.00	72.67	75.37	72.67	73.07
		SG	75.71	70.67	70.78	70.67	69.80
		SG-MeanNor	77.14	72.00	71.08	72.00	69.59
		**SG-DT**	**79.43**	**78.00**	**78.66**	**78.00**	**77.89**
		SG-SNV	79.71	71.33	73.31	71.33	69.06
	KAN	Raw	98.00	91.33	92.82	91.33	91.47
		SG	96.86	92.67	93.43	92.67	92.74
		SG-MeanNor	100	92.67	93.95	92.67	92.75
		**SG-DT**	**97.14**	**94.67**	**95.04**	**94.67**	**94.64**
		SG-SNV	99.71	92.67	93.61	92.67	92.71
SWIR	PLS-DA	Raw (Lvs=14)	82.00	80.00	83.68	80.00	79.21
		SG (Lvs=15)	82.29	82.00	84.82	82.00	81.57
		SG-MeanNor (Lvs=14)	82.29	83.33	86.13	83.33	82.81
		**SG-DT** (Lvs=12)	**87.71**	**85.33**	**86.84**	**85.33**	**85.26**
		SG-SNV (Lvs=11)	84.00	84.67	86.71	84.67	84.20
	SVM	Raw	98.29	93.33	94.38	93.33	93.53
		SG	98.00	94.00	94.98	94.00	94.15
		SG-MeanNor	100	89.33	89.34	89.33	89.22
		**SG-DT**	**100**	**94.67**	**95.04**	**94.67**	**94.74**
		SG-SNV	100	92.00	92.38	92.00	92.03
	MLP	Raw	91.71	94.00	94.64	94.00	93.83
		SG	94.57	95.33	95.40	95.33	95.28
		SG-MeanNor	97.71	94.67	94.84	94.67	94.68
		**SG-DT**	**98.29**	**97.33**	**97.46**	**97.33**	**97.33**
		SG-SNV	95.71	96.00	96.23	96.00	95.98
	CNN	Raw	84.86	81.33	82.31	81.33	81.42
		SG	86.57	82.00	84.61	82.00	82.30
		SG-MeamNor	90.00	84.00	85.90	84.00	84.39
		**SG-DT**	**94.00**	**88.67**	**89.19**	**88.67**	**88.49**
		SG-SNV	90.86	82.00	83.59	82.00	83.59

KAN	Raw	96.00	95.33	95.63	95.33	95.23
	SG	99.71	96.00	96.56	96.00	96.04
	SG-MeamNor	95.14	92.67	93.36	92.67	92.84
	**SG-DT**	**99.71**	**98.67**	**98.75**	**98.67**	**98.64**
	SG-SNV	95.43	96.00	96.19	96.00	96.04

Table S2 Specific characteristic wavelengths selected by different feature selection methods in VNIR

Methods	Nums	Wavelength (nm)
CARS	168	397.66, 402.90, 410.75, 413.37, 416.00, 431.74, 434.37, 437.00, 444.89, 447.52, 450.16, 460.70, 463.34, 465.98, 468.62, 471.26, 473.90, 476.54, 481.83, 484.47, 487.12, 489.77, 492.42, 495.07, 497.72, 500.37, 503.02, 505.67, 508.32, 510.98, 513.63, 529.59, 532.25, 534.91, 537.57, 540.24, 542.91, 545.57, 548.24, 550.91, 553.58, 556.25, 558.92, 561.59, 564.26, 566.94, 569.61, 572.29, 591.04, 593.73, 596.41, 599.10, 601.78, 604.47, 607.16, 609.85, 612.53, 615.23, 617.92, 620.61, 639.48, 642.18, 644.88, 647.58, 650.29, 652.99, 655.69, 658.40, 661.10, 663.81, 666.52, 674.65, 677.36, 680.07, 682.79, 685.50, 688.22, 690.93, 693.65, 696.37, 699.09, 701.81, 704.53, 723.60, 726.33, 729.06, 731.79, 734.52, 737.25, 739.98, 742.72, 750.93, 753.66, 756.40, 759.14, 761.88, 764.62, 767.36, 770.11, 772.85, 775.60, 778.34, 781.09, 786.58, 789.33, 792.08, 794.84, 797.59, 800.34, 808.61, 811.36, 814.12, 816.88, 819.64, 822.40, 825.16, 827.92, 830.69, 833.45, 836.22, 841.75, 844.52, 847.29, 850.06, 852.83, 855.60, 858.37, 869.47, 872.25, 875.03, 877.80, 880.58, 883.37, 897.28, 900.07, 902.86, 905.64, 908.43, 911.22, 914.02, 919.60, 922.39, 925.19, 927.98, 930.78, 933.58, 936.38, 939.18, 944.78, 947.58, 950.38, 953.19, 955.99, 958.80, 961.60, 964.41, 970.03, 972.84, 975.65, 978.46, 981.27, 984.09, 986.90, 989.72, 995.35, 998.17, 1000.99, 1003.81
iVISSA	91	397.66, 400.28, 402.90, 413.37, 416.00, 418.62, 421.24, 423.86, 426.49, 429.12, 431.74, 495.07, 497.72, 500.37, 503.02, 505.67, 508.32, 510.98, 513.63, 516.29, 518.95, 620.61, 623.30, 626.00, 628.69, 631.39, 634.08, 636.78, 639.48, 642.18, 644.88, 647.58, 650.29, 652.99, 655.69, 658.40, 661.10, 663.81, 666.52, 669.23, 671.94, 674.65, 677.36, 680.07, 682.79, 685.50, 688.22, 690.93, 693.65, 718.15, 720.87, 723.60, 726.33, 729.06, 731.79, 734.52, 737.25, 739.98, 742.72, 745.45, 748.19, 750.93, 753.66, 756.40, 759.14, 761.88, 764.62, 767.36, 770.11, 772.85, 775.60, 778.34, 794.84, 844.52, 847.29, 850.06, 852.83, 855.60, 858.37, 861.14, 863.92, 866.69, 869.47, 947.58, 950.38, 953.19, 981.27, 984.09, 986.90, 1000.99, 1003.81
SPA	17	439.63, 447.52, 503.02, 561.59, 734.52, 767.36, 794.84, 866.69, 883.37, 902.86, 930.78, 944.78, 955.99, 989.72, 995.35, 998.17, 1003.81

Table S3 Specific characteristic wavelengths selected by different feature selection methods in SWIR

Methods	Nums	Wavelength (nm)
CARS	27	1056.90, 1102.13, 1105.61, 1143.96, 1147.45, 1154.43, 1157.93, 1168.40, 1171.90, 1175.39, 1210.37, 1217.37, 1220.87, 1266.44, 1269.95, 1273.46, 1276.97, 1280.48, 1312.11, 1393.14, 1417.86, 1435.53, 1439.07, 1499.27, 1527.66, 1541.87, 1684.44
iVISSA	89	949.43, 994.43, 997.90, 1001.37, 1004.83, 1008.30, 1011.77, 1015.24, 1018.71, 1022.18, 1025.65, 1029.12, 1032.59, 1036.06, 1039.53, 1043.00, 1046.48, 1049.95, 1053.42, 1056.90, 1060.38, 1063.85, 1067.33, 1070.81, 1074.29, 1077.77, 1081.24, 1084.72, 1088.20, 1091.69, 1095.17, 1098.65, 1102.13, 1105.61, 1109.09, 1112.58, 1116.06, 1119.55, 1123.03, 1126.51, 1130.00, 1133.49, 1136.99, 1140.47, 1143.96, 1147.45, 1150.94, 1154.43, 1157.93, 1161.41, 1164.91, 1168.40, 1171.90, 1175.39, 1178.89, 1182.39, 1185.88, 1189.39, 1192.88, 1196.38, 1199.87, 1203.37, 1206.87, 1210.37, 1213.87, 1217.37, 1220.87, 1224.37, 1227.88, 1231.38, 1234.88, 1238.39, 1245.40, 1248.91, 1252.42, 1255.92, 1259.43, 1262.94, 1266.44, 1269.95, 1273.46, 1276.97, 1280.48, 1680.87, 1684.44, 1688.02, 1691.59, 1695.17
SPA	16	997.90, 1032.59, 1074.29, 1136.99, 1175.39, 1213.87, 1255.92, 1326.18, 1403.73, 1470.92, 1623.74, 1691.59, 1695.17

Table S4 Classification performance of different models based on wavelength selection methods in the VNIR and SWIR regions

Range	Model	Wavelength selection	nVar	Train (%)	Test (%)			
				Accuracy	Accuracy	Precision	Recall	F1-Score
VNIR	PLS-DA	**CARS** (Lvs=12)	**168**	**82.57**	**84.00**	**85.86**	**84.00**	**81.93**
		iVISSA (Lvs=13)	89	79.14	80.00	81.11	80.00	78.46
		SPA (Lvs=10)	17	75.14	74.67	73.95	74.67	71.94
	SVM	CARS	168	100	89.33	89.98	89.33	89.39
		iVISSA	89	98.57	88.00	89.43	88.00	87.96
		SPA	17	90.00	82.00	83.96	82.00	81.57
	MLP	CARS	168	100	91.33	91.87	91.33	91.31
		iVISSA	89	95.14	89.33	90.05	89.33	88.81
		SPA	17	95.71	90.00	90.70	90.00	89.92
	CNN	CARS	168	80.57	75.33	79.09	75.33	74.52
		iVISSA	89	89.43	76.67	79.06	76.67	76.40
		**SPA**	**17**	**100**	**88.67**	**89.31**	**88.67**	**89.31**
	KAN	**CARS**	**168**	**98.29**	**96.00**	**96.07**	**96.00**	**95.87**
		iVISSA	89	100	88.67	89.62	88.67	88.42
		SPA	17	100	90.67	90.73	90.67	90.61
SWIR	PLS-DA	CARS (Lvs=13)	27	80.57	78.67	81.88	78.67	75.53
		iVISSA (Lvs=11)	89	84.29	82.67	84.25	82.67	81.09
		SPA (Lvs=10)	16	84.29	80.67	81.40	80.67	78.65
	SVM	CARS	27	95.14	86.67	86.93	86.67	86.43
		iVISSA	89	86.00	90.00	89.33	90.00	89.87
		SPA	16	96.29	90.67	91.43	90.67	90.59
	MLP	CARS	27	90.57	91.33	91.43	91.33	91.24
		iVISSA	89	88.86	90.00	90.52	90.00	89.85
		SPA	16	96.86	95.33	95.77	95.33	95.13
	CNN	CARS	168	93.14	92.00	92.35	91.95	92.35
		iVISSA	89	80.29	81.33	82.53	81.33	81.30
		**SPA**	**17**	**99.74**	**94.00**	**94.13**	**94.00**	**93.40**
	KAN	CARS	27	94.29	90.67	91.75	90.67	90.81
		iVISSA	89	94.57	92.67	92.66	92.67	92.56
		**SPA**	**16**	**99.14**	**98.67**	**98.75**	**98.67**	**98.64**

Table S5. Stratified 5-fold cross-validation performance of selected models.

Model	Accuracy (%)	Precision (%)	Recall (%)	F1-score (%)
SWIR-SG-DT-PLS-DA	83.60 ± 3.21	84.79 ± 2.34	83.60 ± 3.21	83.05 ± 3.45
SWIR-SG-DT-SVM	86.40 ± 3.21	87.82 ± 3.49	86.40 ± 3.21	85.99 ± 3.47
SWIR-SG-DT-MLP	95.80 ± 1.30	96.25 ± 1.22	95.80 ± 1.30	95.77 ± 1.36
SWIR-SG-DT-CNN	86.60 ± 3.05	87.60 ± 2.79	86.60 ± 3.05	86.59 ± 2.89
SWIR-SG-DT-KAN	96.00 ± 1.41	96.35 ± 1.38	96.00 ± 1.41	95.98 ± 1.45
SWIR-SG-DT-SPA-KAN	97.40 ± 1.82	97.58 ± 1.68	97.40 ± 1.82	97.40 ± 1.81

(a)

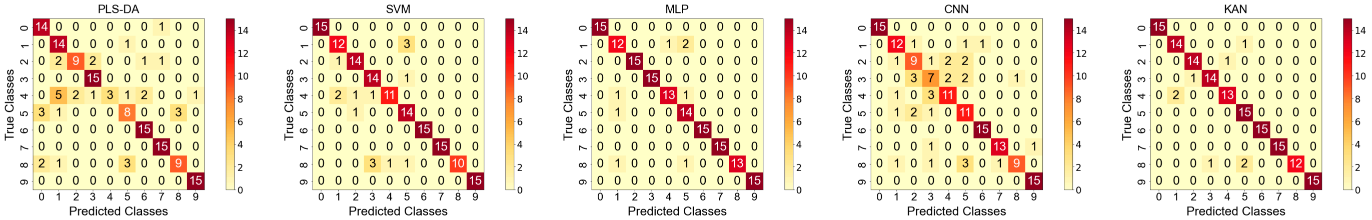

(b)

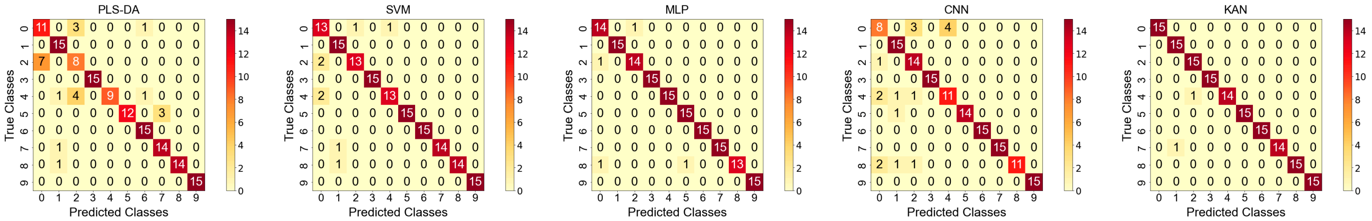

Figure. S1. Classification performance of different models using full-spectrum data with optimal preprocessing. (a) VNIR, (b) SWIR

Note: 0: Butterfly fish maw; 1: Douhu Jiao; 2: Egg fish maw; 3: Dried fish maw; 4: Zuoluo Jiao; 5: Baihua Jiao; 6: Beihai Jiao; 7: Dakou jiao; 8: Zhuye Jiao; 9: Zhizhu Jiao.
